# Factors Influencing Length of Stay of Forensic Patients: Impact of Clinical and Psychosocial Variables in Medium Secure Setting

**DOI:** 10.3389/fpsyt.2020.00810

**Published:** 2020-08-14

**Authors:** Paweł Gosek, Justyna Kotowska, Elżbieta Rowińska-Garbień, Dariusz Bartczak, Janusz Heitzman

**Affiliations:** ^1^ Department of Forensic Psychiatry, Institute of Psychiatry and Neurology, Warsaw, Poland; ^2^ Chair of Psychiatry, Medical College, Jagiellonian University, Krakow, Poland; ^3^ Sexological Clinic and Pathology of Intercourse, The Nowowiejski Hospital, Warsaw, Poland

**Keywords:** forensic psychiatry, violence, length of stay, treatment resistance, forensic treatment

## Abstract

**Objectives:**

Forensic psychiatric care has two, often contradictory, aims—the treatment of mentally ill offenders and the isolation of the perpetrators to ensure public safety. It is essential to ensure that any periods of liberty deprivation do not last longer than necessary to provide appropriate treatment. Therefore, identifying the factors affecting the length of stay (LoS) is one of the most important research areas in the forensic psychiatry. The literature on this subject is scarce and to date there no data available on LoS for patients in Eastern or Central European patients.

**Methods:**

We conducted a retrospective analysis of data for 150 inpatients in a medium secure unit. Based on a literature review and clinical experience, variables potentially influencing LoS were identified and included in the analysis.

**Results:**

The variables that were significantly associated with LoS included duration of mental illness; severity of index offense; whether a crime was committed as a result of hallucinations or during drug treatment discontinuation; if the index offenses was a continuous crime (crimes committed over an extended period of time); persistent psychosis; multiple antipsychotic treatments; as well as a diagnosis of schizophrenia and schizoaffective disorder.

**Conclusions:**

Our findings are highly consistent with observations made by other researchers. However, contrary the majority of previous studies our project incorporates data concerning the clinical presentation of patients. For example, we demonstrate that variables measuring treatment resistance might be one of the crucial determinants of LoS, which is a novel research finding.

## Introduction

Forensic psychiatric care has to meet two, often contradictory, expectations—treatment of mentally ill offenders and their reintegration with the society, as well as isolation of perpetrators and ensuring public safety. The involuntary nature of this care with its loss of personal liberty has to be carried out with full respect to human rights and should not exceed what is absolutely necessary. Most European countries allow for forensic inpatient treatment, sometimes called “psychiatric detention”, which often exceeds the maximum length of a prison sentence that would be adjudicated for similar offenses committed by healthy perpetrators (1). This poses a risk of disproportionately long and protracted stays in forensic institutions. Alternatively, unstable patients may be prematurely discharged, which may lead to worse overall outcomes, poorer quality of life, and increased violence and re-admission risk. Therefore, forensic inpatients’ length of stay (LoS) and factors affecting LoS are of special interest among forensic professionals. In countries where dedicated long-stay services exist, the percentage of long-stay patients (usually defined as a period of treatment >5 years) was estimated to be 15–20% (2). The identification of specific features of therapy and treatment duration that may result in optimized care, better risk assessment, and more appropriate management may lead to improved quality of care and patients’ quality of life.

Internationally, admission rates to forensic institutions are rising among most European countries (3, 4), with a few exceptions (5). Data on average LoS covering is scarce, but some authors suggest that LoS is also increasing (6, 7). Factors associated with LoS of forensic inpatients may be attributable to clinical patient characteristics, including the course of disease and severity of symptoms, compliance with previous treatment, available social and family support, and a history of aggression or criminal involvement. In addition, external factors like the unique characteristics of each European country’s judicial system, criteria for admission, amount of resources for general psychiatric care staff, and social and community support may also influence LoS (8).

In European countries, several research projects have been conducted to evaluate LoS of forensic inpatients. Ross et al. (9) compared a group of patients detained for under 4 years with a group detained for over 10 years in a German population. Age at first admission, type of offense, living situation at the time of offense, immigration status, patient’s employment before admission, and being sentenced to prison prior to the admission were identified as predictors of a short or long stay at forensic psychiatry institutions. In a Swedish population, Andreasson et al. (10) found that previous contact with child and adolescent psychiatric services, violent index offenseds, psychotic disorders, a history of substance abuse, and absconding during treatment predicted longer LoS. In the United Kingdom, among patients detained in medium secure units, diagnosis of a psychotic disorder, previous multiple admissions, detention under a hospital order, being on a restriction order, and a history of moderately violent crimes were associated with prolonged LoS (11).

In a subsequent UK study on a sample consisting of patients from high and medium secure units, Völlm et al. (12) described the characteristics of long-stay patients (defined as >5 years in medium secure care, >10 years in high secure care, or >15 years in both), based on file reviews of 401 inpatients. The majority of these patients (57.9%) were diagnosed with schizophrenia and 32.8% were considered to be treatment resistant. In comparison to the general forensic psychiatric patient population the authors found that the percentage of personality disorders was much higher among the group of long-stay patients. Most individuals in the long-stay sample were classified as primarily severely violent offenders and the most common type of offense were offenses against the person, followed by sex offenses and property offenses. The proportion of sexual offenses and arsons as index offenses appeared to be higher than those reported in the general forensic population. A substantial proportion of these long-stay patients committed an offense within an institutional setting, and over a quarter seriously assaulted a staff member within the past 5 years. Forty-four per cent had been in seclusion and 12% had serious self-harm episodes during the past 5 years.

In the Republic of Ireland, a subgroup of long-stay patients was more likely to be charged with serious violence and to suffer from psychotic disorders (13). A naturalistic prospective cohort study by Davoren et al. (14) of an Irish population of 279 forensic inpatients reported sociodemographic data, DUNDRUM Toolkit scores (15), and HCR-20 scores. The authors found that male gender, most items on the DUNDRUM-1 scale (with the exception of suicide-related items and the need to prevent access to weapons, drugs, or media), item 1 “location” on the DUNDRUM-2 scale, item H1 “past violence” and item H2 “young age at first violence incident” from HCR-20 predicted longer LoS. Conversely, having no mental disorder other than an adjustment disorder, item H9 “personality disorder” and C2 “negative attitudes” from the HCR-20 predicted shorter LoS. Being found not guilty by reason of insanity or being detained under civil mental health legislation tended to predict longer lengths of stay. One finding of special interest in this study was that neither episodes of harm to others nor need for seclusion during admission predicted LoS.

In a Dutch sample of 139 inpatients of long-term forensic psychiatric care (n = 61) and regular forensic psychiatric care (n = 78) (16), patients staying in long-term facilities were more often born in a Dutch Caribbean country, less often had a substance abuse disorder, were more often emotionally neglected during childhood, had a higher HCR-20 risk item score, a higher security needs score, a higher (meaning less successful) recovery score, recidivated more frequently, and had absconded more often than patients in regular forensic care.

While the results of the research on LoS are consistent for some factors, the literature also presents factors for which evidence is mixed, including substance abuse (10, 11, 16, 17) and diagnosis (9–11, 17). So far, no studies have been conducted that have investigates LoS in Eastern or Central European populations.

It is difficult to compare research findings for forensic psychiatric populations across European countries due to the significant differences that exist between them. One essential step that can assist in making such comparison, however, is to precisely define the study group. Polish forensic psychiatric inpatient care (18) is a three-step system composed of high, medium, and low security units. The legal framework allows for the detention of insane perpetrators at the time of the criminal act or of those with diminished criminal responsibility due to a mental disorder in cases of severe crimes. For insane offenders [defined as an individual who: “at the time of the commission of a prohibited act, was incapable of recognizing its (the act’s) significance or controlling their conduct because of a mental disease, mental deficiency, or another mental disturbance] for which there has been an assessment concluding that this individual poses a high risk of reoffending, it is possible to apply one of four preventive measures including involuntary placement and treatment in a forensic institution (*psychiatric* *forensic detention*).

Individuals found to be insane usually suffer from psychosis, intellectual disability, dementia, or another serious mental disorder. The duration of the preventive measure is not predetermined. Placement in a psychiatric institution is possible only when it’s necessary to prevent reoffending that causes severe social or physical harm, and other legal measures are not sufficient to achieve this goal. Out-patient forensic treatment is also possible. The total number of forensic psychiatric beds has varied over the years, but is currently approximately 3,000 in over 50 forensic institutions across the country. The Board for Preventive Measures, an institution under the direct control of the Ministry of Health, provides a central mechanism for allocating inpatients, appointing the level of security for patients starting their detention, ruling on the prolongation of treatment, and making recommendations to competent courts regarding discharge or transfer.

The aim of our study was to identify the factors associated with LoS in a medium secure inpatient forensic psychiatric care setting in Poland. As mentioned above, due to the significant variety of forensic psychiatric care models in Europe, direct country-to-country comparisons are difficult. Optimal conditions for comparability will be achieved by comparing data from our study with data from countries with similar forensic psychiatric service organization, such as a three-step model of care, with a similar rate of concomitant substance misuse, possible outpatient forensic psychiatric care, and criteria for admissions as described by Salize et al. (19, 20). Considering an upcoming reform of the Polish forensic psychiatry system and the need to implement common guidelines of care (21), the data presented could constitute an important, evidence-based consideration which is the first of this scale in Poland that could be taken into account when reshaping forensic psychiatric care in Poland and other Central and Eastern European countries.

## Method

### Data Collection

An electronic database was developed to document the characteristics of the study sample and the potential factors influencing LoS, including, among others, socio-demographic data, course of mental disorder, previous contacts with psychiatric care services, history of aggressive and self-destructive behavior, current mental health condition, length of stay in the previous forensic admissions, and criminal data e.g. severity of the criminal act, time spent in custody. The list of potential factors affecting LoS was developed by the research team of three experienced psychiatrists and two experienced forensic psychologists, based on the literature review (described briefly above) and based on clinical experience. Data were entered into the electronic system by trained psychologists and psychiatrists who were involved in clinical work in the medium security department. The database contained 63 variables for each of the 150 patients. The source of data included current and past medical records, psychiatric and psychological experts’ opinions concerning the sanity evaluation at the time of the criminal act, periodic psychiatric and psychological experts’ opinions issued every 6 months in the course of inpatient treatment. Reliability checks were performed before the statistical analysis. Reliability checks included the verification of data from source materials and database entries regarding personal data and five randomly selected variables for each subject, which were then performed by a project researcher not involved in the collection of data on that topic. Data were verified using available source documentation. For the purpose of the current study, variables identified as potentially influencing LoS were extracted and analyzed.

### Sampling

The study group consisted of 150 inpatients (123 males and 27 females) referred by the court and admitted between 01.01.2014 and 31.12.2018 to the medium security forensic inpatient center at the Department of Forensic Psychiatry of the Institute of Psychiatry and Neurology in Warsaw (DFP IPIN). Patients were admitted for involuntary treatment in a forensic institution (people found not guilty due to insanity or those with diminished criminal responsibility). The analysis included data for all patients admitted and hospitalized within this time period. There were no exclusion criteria.

Before committing the prohibited criminal act most of the subjects lived in an urban area, received social benefits or were unemployed. Mean age at time of admission was 40.07 years (SD: 12.99), mean age at the time of the study was 43.38 years (SD: 13.41). Mean LoS was 39.14 months (SD: 42.45, M = 21.5 months) and the mean LoS in DFP IPIN was 21.11 months (SD: 15.12, M = 18 months). The study group consisted mostly of subjects suffering from psychotic disorders, among which schizophrenia (n = 86, 55.3%), organic mental disorders (n = 17, 11.3%), and delusional disorder (n = 16, 10.6%) were the most prevalent. Categorical diagnoses based on ICD-10 were used as these are standard practice in clinical settings in Poland. The analysis of legal documentation including court proceedings and psychiatric evaluation reports revealed that the most prevalent criminal act was homicide and attempted homicide (n = 47, 31.3%), followed by threats of harm (n = 30, 20%), and serious bodily injury (n = 23, 15.3%). As regards the victims, a majority of criminal acts related to patients’ family members, including parents (n = 38, 25.3%), siblings (n = 14, 9.3%), partner/wife/husband (n = 13, 8.6%), children (n = 9, 6.0%), and other family members (n = 5, 3.3%). Acquaintances and strangers were reported in 42.6% of cases (n = 64). A minority of criminal acts were committed against property and public safety, 1.3 and 3.3% respectively. At the end of the study period, 42% of subjects (n = 62) resided at DFP IPIN. Forty-four per cent of subjects were transferred to low secure units (n = 66), 5% (n = 7) were transferred to other medium secure units, 7% (n = 11) to one of the three regional maximum-security hospitals, two subjects died due to a somatic illness, and two were discharged directly home. It should be assumed that the overall LoS for most of the subjects is significantly longer than reported in this study as treatment for most patients did not end before or at the end-point of the study.

The characteristics of the study group is presented in **Table 1**.

**Table 1:** Characteristics of the study group.

Variables		Number	Percentage	Mean	Range	SD
*Total number of patients*		150	100%			
*Demographic data*						
	Age, years			43.38	21–81	13.41
	Sex, female	27	18%			
	Sex, male	123	82%			
*Residence status before the index offense*						
	Rural area	36	24%			
	City area	114	76%			
*Employment status at the time of offense*						
	Employed	15	10.0%			
	Unemployed, no work experience	49	32.6%			
	Unemployed, work experience	34	22.7%			
	Receiving social benefits	52	34.7%			
*Highest educational status achieved*						
	Assisted primary (special needs)	12	8.0%			
	Primary (9 years of education)	42	28.0%			
	Secondary (12 years of education)	82	54.7%			
	University	14	9.3%			
*Diagnosis at admission*						
	Schizophrenia	86	57.3%			
	Organic mental disorders	17	13.3%			
	Delusional disorders	17	13.3%			
	Schizoaffective disorder	8	5.3%			
	Intellectual disability	8	5.3%			
	Drug induced psychosis	7	4.7%			
	Personality disorders	5	3.3%			
	Affective disorders (Bipolar Disorder, Major Depressive Disorder)	2	1.3%			
*Treatment pathway*						
	Transferred to low security unit	66	44.0%			
	Current stay	62	41.3%			
	Transferred to high security unit	11	7.3%			
	Transferred to medium security unit	7	4.7%			
	Discharged home	2	1.3%			
	Died	2	1.3%			
*Characteristic of the criminal act*						
	Homicide/attempted homicide	47	31.3%			
	Serious threats	30	20.0%			
	Serious body injury	23	15.3%			
	Bulling	20	13.3%			
	Sexual offenses	17	11.3%			
	Others	21	14%			
*Age at the beginning of forensic treatment (years)*				*40.07*	*19–75*	*12.99*
*Length of stay in current unit (months)*				*21.11*	*1–57*	*15.12*
*Length of forensic treatment (months)*				*39.14*	*1–211*	*42.45*

### Statistical Analysis

A statistical analysis was performed for each variable hypothesized to be associated with LoS. Variables were coded dichotomously, for example as present/absent etc. Statistical methods were chosen depending on the measurement level and distribution of the data. For all continuous variables, the Lillefors test was performed. None of the reported variables were normally distributed (p < 0.01). Therefore, non-parametric tests were used including the Mann-Whitney U Test and Kruskal-Wallis Test. In regression modeling, the generalized linear model (GLM) was used as variables were not normally distributed.

### Ethical Approval

In accordance with Polish legal regulations the relevant Ethical Commission of the Institute of Psychiatry and Neurology in Warsaw was informed about the project design.

## Results

**Table 2** presents the associations of the dichotomously-measured variables and LoS. Demographical variables including sex, living area before treatment (rural or urban), historical data on the course of the mental disorder, e.g. mental disorder diagnosed among family members, concomitant alcohol/psychoactive substance dependence, psychiatric treatment in the past, were not significantly related to LoS. Number of past admissions to forensic units (first or second admission) and patient’s age at admission were also not significantly associated with LoS.

**Table 2:** Factors influencing length of stay.

Variables		N = 150	U-Mann Whitney Test						
			Rank 1n = (%)	Rank 2n = (%)	Sum of rank 1	Sum of rank 2	U value	Z value	p
		Demographical and clinical factors							
Sex male/female			123 (82.0)	27 (18.0)	9294,500	2030,500	1652,500	0,036688	0,970
Place of residence urban/rural			115 (76.7)	35 (23.3)	8421,000	2605,000	1975,000	0,009025	0,992
Mental disorders among family members no/yes			119 (79.3)	31 (20.7)	9152,000	2023,000	1527,000	1,409972	0,158
Alcohol dependence no/yes			78 (52.0)	72 (48.0)	6253,500	5071,500	2443,500	1,369273	0,171
Drugs/psychoactive substance/legal highs abuse no/yes			103 (68.7)	46 (31.3)	7992,000	3183,000	2102,000	1,095077	0,273
Diagnosis of intellectual disability in the past no/yes			133 (88.7)	17 (11.3)	9809,500	1068,500	915,5000	1,144789	0,252
Regular (systematic) psychiatric treatment in the past no/yes			138 (92.0)	12 (8.0)	10527,00	798,0000	720,0000	0,744698	0,456
	Characteristics of the offense								
Characteristics of the offense single/continuing			95 (63.3)	55 (36.7)	7660,500	3664,500	2124,500	1,901225	0,057
Offense previously planned or prepared no/yes			98 (65.3)	52 (34.7)	7552,500	3772,500	2394,500	0,604197	0,546
Offense under influence of alcohol/psychoactive substances no/yes			80 (53.3)	70 (46.7)	6446,000	4879,000	2394,000	1,527563	0,127
Offense under influence of delusions no/yes			34 (22.7)	116 (77.3)	2574,000	8751,000	1965,000	0,029177	0,977
Offense under influence of hallucinations no/yes			91 (60.7)	59 (39.3)	6279,500	5045,500	2093,500	-2,27183	0,023
Offense in the course of medicine discontinuation yes/no			93 (62.0)	57 (38.0)	7590,000	3735,000	2082,000	2,199235	0,028
	Clinical presentation in the last 6 months								
Aggressive behavior no/yes			117 (78.0)	33 (22.0)	8940,000	2385,000	1824,000	0,480904	0,631
Self-destructive behavior no/yes			139 (92.7)	11 (7.3)	10667,00	658,0000	592,0000	1,240015	0,215
Persistent psychotic symptoms no/yes			98 (65.3)	52 (34.7)	6814,000	4511,000	1963,000	-2,30819	0,021
Treatment with >1 antipsychotics no/yes			69 (46.0)	81 (54.0)	4119,000	7206,000	1704,000	-4,11019	0,000
Clozapine treatment no/yes			141 (94.0)	9 (6.0)	10523,50	801,5000	512,5000	-0,961496	0,336
			Spearman’s correlation						
			Mean	Range			R Spearman	t(N-2)	p
Age (years) at the admission to forensic unit							0,107795	1,319064	0,189185
Number of admission to forensic unit				1–2			-0,049361	-0,601231	0,548606
Duration of mental illness (number of years from onset)							0,326626	4,204159	0,000045

On the contrary, the duration of mental disorder, defined as the number of years since the onset of the mental disorder, was associated with the prolongation of LoS (p = 0.000045). Offenses committed in response to hallucinations (p = 0.02) or in the course of medicine discontinuation (p = 0.02) were associated with longer LoS. Among the variables describing the clinical and behavioral presentation of patients in the past 6 months (prior to transfer to another unit, discharge, or admission), persistent psychotic symptoms (p = 0.02) and treatment combined with at least two antipsychotics (p = 0.03) were associated with longer LoS. As regards diagnosis, the results suggest that diagnoses of schizophrenia or schizoaffective disorder were most closely associated with LoS (**Table 3**). The index offenses most closely associated with LoS was homicide/attempted homicide and threats of harm (**Table 4**).

**Table 3:** Influence of diagnosis on length of stay.

Dependent variable:	Kruskal-Wallis one-way analysis of variance by ranks (ANOVA) Independent variable: diagnosis Kruskal-Wallis Test: H (8, N = 150) = 24,66962 p =,0018		
Length of stay in forensic institutions	N	Sum of ranks	Mean rank
Schizophrenia	86	7401,500	86,0640
Intellectual disability	8	383,500	47,9375
Organic mental disorder	17	879,000	51,7059
Delusional disorder	16	1085,500	67,8438
Substance use psychosis	7	356,500	50,9286
Personality disorders	5	303,000	60,6000
Affective disorders (Bipolar Disorder, Major Depressive Disorder)	2	77,500	38,7500
Schizoaffective disorders	8	837,000	104,6250
Persistent delusional disorder	1	1,500	1,5000

**Table 4:** Influence of the type of criminal offense on length of stay.

Dependent variable: Length of stay in forensic institutions	p value calculated for multiple (bilateral) comparisons length of stay in forensic institutionsindependent variable: type of crime; Kruskal-Wallis Test: H (4, N = 129) = 12,13200 p =,0164				
	Homicide/homicide attempt R:78,415	Sexual offenses R:60,029	Bullying R:54,175	Threats of harm R:48,909	Serious body injury R:66,065
Homicide/homicide attempt		0,822583	0,151513	0,022476	1,000000
Sexual offenses	0,822583		1,000000	1,000000	1,000000
Bullying	0,151513	1,000000		1,000000	1,000000
Threats of harm	0,022476	1,000000	1,000000		1,000000
Serious body injury	1,000000	1,000000	1,000000	1,000000	

Due to the non-normal distribution of the variables, regression was conducted using a generalized linear model (GLM) approach. Only variables that were significantly associated with LoS in direct comparisons were included. The regression revealed that persistent psychotic symptoms (p = 0.003) and treatment combined with at least two antipsychotics (p = 0.001) significantly predicted LoS. The generalized linear model is presented in **Table 5**.

**Table 5:** Generalized linear model - length of stay in forensic institutions.

Explanatory variables	B	SE	95% Confidence Interval		Significance level		
			Lower	Upper	χ2 Wald	df	p
Offense under influence of hallucinations	5.845	6.5693	-18.721	7.030	0.792	1	0.374
Offense in the course of medicine discontinuation	8.344	6.6328	-21.344	4.656	1.583	1	0.208
Treatment with >1 antipsychotics	21.299	6.5058	-34.050	-8.548	10.718	1	0.001
Persistent psychotic symptoms	20.105	6.8461	-33.523	-6.687	8.624	1	0.003

## Discussion

The data in this study describe roughly 5% of the Polish forensic psychiatric population. The mean LoS in this sample was 39.15 months (SD 42.46), median 25.5 months. As there are no published data on the average LoS in forensic institutions in Poland or in Eastern European countries, we cannot compare our results with those of similar forensic service providers. Moreover, there are no data concerning the number of long-stay patients, and due to the lack of any national register, there are also no annual data on the exact number of patients discharged from forensic institutions.

The main aim of the study was to evaluate the associations between several variables and LoS in a medium security hospital. As our study was retrospective and data were collected for patients admitted to the hospital within a five-year timeframe, the variables were tested for then staying patients or for patients being under discharge or transfer to another service units at that time, we are aware that the average LoS in forensic institutions in Poland is longer than that reported for the study group. Inpatient forensic psychiatric care in Poland is a three-stage system with high, medium, and low security hospitals. The allocation of a patient to a particular level of security is primarily based on the type of index offense committed. However, previous area of residence and number of available beds are also considered by the Board for Preventive Measures, directed by the Ministry of Health, when making this decision. To avoid a potential bias deriving from the overrepresentation of perpetrators who committed less severe crimes and were directly allocated to low secure units, we decided to include only inpatients in medium secure units in the study.

Among the socio-demographical factors investigated in this study, we found that neither sex nor the place of residence were associated with LoS. In most European countries the proportion of male forensic psychiatric patients typically ranges from 80–90% (10, 11, 22), which is similar to the data presented in this study. Davoren et al. found that being male predicted a longer stay in forensic care (14), but we did not observe such a relationship. In Poland, there are no high security forensic psychiatric institutions for females. Therefore, we hypothesise that the lack of a significant finding in relation to sex in our study might be related to the overrepresentation of female perpetrators who have committed the most severe crimes in our sample.

Diagnoses of mental disorder among family members and a diagnosis of intellectual disability did not significantly predict LoS in the study group. Previous contact with child and adolescent psychiatric services, reflecting in some way the duration of illness, was previously described as one of the important factors of LoS prolongation (10). As we have not been able to access the detailed medical registers for each of the study participants, we analyzed the overall duration of the disease, defined as the number of years since the onset of the mental disorder. A clear association with LoS prolongation was observed. As our sample consisted of mostly psychotic patients, a possible explanation of this finding could pertain to the negative impact of persistent psychosis on cognitive functioning, social cognition, and decision-making processes observed in patients suffering from psychosis (23, 24).

Some studies indicate that alcohol or substance misuse may increase LoS in forensic settings (10), but other studies found the opposite (11, 14). In our sample, 48% of participants were alcohol dependent and 31.3% were diagnosed with other substance misuse. We observed no statistically significant relationship with LoS. Similarly, no prolongation of LoS was observed with respect to offenses committed under the influence of alcohol or another substance. These non-signifiacnt findings may be related to the availability of therapeutic methods in our department, including specifically treatment for substance use which is extensive and easily accessible. The verification of these results requires testing in a wider population across multiple treatment sites.

Our sample consisted mostly of perpetrators of severe crimes. Homicide, attempted homicide, and serious bodily injury constituted almost half of the cases. The Kruskal-Wallis Test revealed a significant relationship between the of severity of the criminal behavior (homicide or attempted homicide) on LoS. This is consistent with earlier observations by Ross et al. (9), Andreasson et al. (10) and Völlm et al. (12). In addition to several clinical factors influencing LoS on general psychiatric wards (25), in forensic psychiatric care one of the key issues taken into consideration at time of discharge is risk assessment. In Poland, the court responsible for the termination or prolongation of forensic detention requires that periodic reports referring to current mental state, the risk of relapse, and the risk of reoffending are produced for each patient. Risk is assessed with regard to self-harm and risks to society. The severity of the index offense typically pertains to risk posed to others. Therefore, the severity of an earlier offense might sensitize clinicians to the possibility of reoffending who then extend LoS.

An important aspect of the study was retrospective, based on medical records containing data from court proceedings. We evaluated the mental state of perpetrators at the time of committing a criminal offense. In the case of perpetrators of non-continuous crimes, defined as lasting no longer than hours (contrary to continuous crimes lasting days or longer, for example bullying or recurring threats), a statistical trend (p = 0.057) to prolong LoS was observed. No significant differences were observed regarding whether the criminal act was planned or not, committed under the influence of alcohol or psychoactive substances, or as a result of delusions. According to Appelbaum et al. (26) while the occurrence of delusions may intensify aggressive behaviors, it does not affect the overall risk of aggression in this group of patients. Similarly, the results of a study by Junginger et al. (27) indicate that a delusional motivation behind aggressive behavior is uncommon. In our sample, the majority of subjects committed a crime as a result of delusions (n = 116 of 150). Data from previous studies of Junginger et al. (28, 29) indicates that the risk of aggressive behavior is increased by the presence of command hallucinations. Command hallucinations occurred at the time of the criminal act in 59 of 150 subjects. Criminal acts committed under the influence of hallucinations were associated with prolonged LoS (p = 0.023). A possible explanation of this phenomenon could be related to the discontinuation of antipsychotics at the time of the criminal act or drug resistance. In our sample, subjects who committed a crime in the course of medicine discontinuation in forensic institutions spent more time in forensic units than the subjects who had good antipsychotic medication compliance (p = 0.027). We could not measure the relationship between treatment resistant psychosis and LoS as this was not recorded in the medical documentation analyzed for this study. However, the results mentioned previously, and the clinical factors described later in this paper suggest that treatment resistance could be an important factor for LoS prolongation.

There is a risk of bias when retrospectively evaluating mental state and results need to be interpreted carefully. However, the process of forensic psychiatric assessment during the court proceedings should be very detailed and incorporate all possible evidence concerning the behavior of an individual, including for example witness statements and medical records. Further, in the case of severe crimes and where there exist any doubts regarding the mental state of perpetrators it is a common practice in the Polish legal system that perpetrators are observed in a clinical psychiatric department for at least 4 weeks. This is a legal requirement from the courts. In light these assessments, the courts decide whether individuals are healthy or mentally ill and determine the sentence or treatment order. Typically, at least two independent, experienced psychiatrists have to issue an expert opinion concerning the mental state of a perpetrator, and in the case of severe crimes, the number of expert opinions increases. As we collected data from current medical records and from court proceedings, the risk of bias deriving from the retrospective design of the study is minimized.

Among clinical factors, a diagnosis of schizophrenia and schizoaffective psychosis was one of the most prominent factors associated with LoS. In the one-way analysis of variance from among eight categorical diagnoses based on the ICD-10, schizophrenia and schizoaffective disorder were found to significantly prolong LoS [Kruskal-Wallis Test: H (8, N = 150) = 24.66962 p = .0018], which is consistent with earlier studies (10–13). The findings deriving from our sample failed to confirm the observations of Chester et al. (30) that patients diagnosed with an intellectual disability had significantly shorter stays in forensic institutions, however, conclusions pertaining to this group are limited by a small number of these patients in our sample. There is good evidence to support an association between violence and schizophrenia (31, 32), and a systematic review and meta-analysis of 128 studies (33) found higher rates of inpatient violence in forensic settings compared to acute psychiatric wards in each of 10 countries surveyed.

Moreover, mentally disordered offenders in forensic psychiatric settings are also at a greater risk of suicide in comparison with the general population (34). It was initially surprising that in our sample neither aggressive nor self-destructive behavior observed during the last 6 months of stay prolonged LoS. However, this was also reported in the study by Davoren et al. (14). The number of aggressive events including assaults on staff and auto-aggressive behavior was low in our sample in comparison with other samples (12), which may be due to our shorter study timeframe. Another possible explanation is that in clinical practice the majority of aggressive individuals are transferred to higher security departments, therefore, as the protocol of the study does not include a follow-up period, it is highly probable that the overall LoS of those individuals will be extended in the future.

In general psychiatric settings, current mental state, including the presence of severe psychotic symptoms, is one of the main factors affecting discharge decisions. In our study, based on the analysis of medical records and reports issued to courts, we evaluate the current (at the time of transfer to other hospitals for transferred subjects or at the time of enrollment to the study for current inpatients) presence of persistent psychotic symptoms and the pharmacological treatments used (single or multiple antipsychotic medication). These two variables proved to be important factors associated with prolonging patients’ length of stay (respectively p = 0.02 and p = 0.00004). Both polypharmacotherapy and persistent psychotic symptoms, despite adequate medical treatment, are indicators of drug resistant psychosis. Similarly, in a UK sample (12), over 30% of long-stay patients suffering from schizophrenia were considered to be treatment resistant. Another factor that may relate to treatment resistance in clinical settings is the use of clozapine. In our sample use of clozapine variable was not significantly correlated to LoS, however the number of subjects receiving clozapine was quite low (n = 9). We cannot rule out the possibility that clozapine has been badly tolerated by some of the subjects in the past, which could be the reason for using another medication currently, despite treatment resistance. Longstanding persistence of psychotic symptoms may impede cognitive functions and social cognition, leading to the lack of insight, which can increase the risk of violence (23, 24).

In conclusion, our results support some previous findings concerning factors relating to the LoS of inpatients in forensic psychiatric settings in an East European sample. The severity of criminal acts proved to be one of the most significant factors related to LoS prolongation, as well as diagnoses of schizophrenia and schizoaffective disorder. The impact of diagnosis should be interpreted carefully however as any comparisons with results from the international literature is at risk of bias as the criteria for admission to forensic institutions and the profiles of the patient populations differ significantly across European countries (19, 20). Contrary to most of the previous reports, our study incorporates data concerning the clinical presentation of subjects during forensic treatment. Our analysis of the data on mental state at the time the index offense, mental state at the time of the study, and the treatment offered in the course of psychiatric detention indicates that one of the crucial factors prolonging LoS is treatment resistance. The significance of treatment resistance is a novel finding and has not been discussed in earlier publications on this topic. Our findings may significantly affect day-to-day clinical practice. Findings suggest that optimizations of the medical approach may prevent long hospital stays and lengthy deprivations of liberty.

### Limitations

The obvious limitation of our study is the retrospective and cross-sectional design. The research protocol included collecting two types of data: medical records and data from court proceedings. The data was based on at least two independent assessments of trained professionals, psychiatrists, and psychologists during court proceedings and at least once during the current hospital stay for each subject. Such an approach is uncommon in standard clinical settings and minimizes the risk of bias resulting from the retrospective design. Conclusions derived from our study should not be generalized to the whole population of forensic inpatients in Poland as data pertain to a medium secure setting. We cannot rule out that the relationships between specific variables and LoS could be different in low secure settings, due to the differences in patient populations. As forensic inpatient care in Poland is a three-step model with units of high, medium, and low security, the total length of stay of forensic patients in Poland is likely longer than that reported in this study and it should be assumed that it will be longer for patients transferred to wards within a maximum security hospital. The cross-sectional design of the study rules out the assessment of the total LoS.

## Data Availability Statement

The datasets generated for this study are available on request to the corresponding author.

## Ethics Statement

Ethical review and approval were not required for the study on human participants in accordance with the local legislation and institutional requirements. Written informed consent for participation was not required for this study in accordance with the national legislation and the institutional requirements.

## Author Contributions

PG—Concept of project, literature search, database preparation, data collection, statistical analysis, manuscript editing, final text approval. JK—Literature search, database preparation, data collection, statistical analysis, manuscript editing. ER-G—Database preparation, data collection. DB—Database preparation, data collection. JH—Concept of project, manuscript editing, final text approval.

## Conflict of Interest

The authors declare that the research was conducted in the absence of any commercial or financial relationships that could be construed as a potential conflict of interest.
